# Electro-acupuncture for health-related quality of life and symptoms in patients with gastric cancer undergoing adjuvant chemotherapy (EAGER): a protocol for a multicenter randomized controlled trial

**DOI:** 10.1186/s12955-023-02135-9

**Published:** 2023-07-11

**Authors:** Xuesong Chang, Yanjuan Zhu, Wenjie Zhao, Yihong Liu, Yihan He, Yadong Chen, Danghan Xu, Jun J. Mao, Hai-bo Zhang

**Affiliations:** 1 Department of Oncology, Guangdong Provincial Hospital of Traditional Chinese Medicine, the Second Affiliated Hospital of Guangzhou University of Chinese Medicine, Guangzhou, China; 2 Guangdong Provincial Key Laboratory of Clinical Research on Traditional Chinese Medicine Syndrome, Guangzhou, China; 3 Guangdong-Hong Kong-Macau Joint Lab on Chinese Medicine and Immune Disease Research, Guangzhou University of Chinese Medicine, Guangzhou, China; 4 The Second Clinical Medical School, Guangzhou University of Chinese Medicine, Guangzhou, China; 5 Rehabilitation Center, the First Affiliated Hospital of Guangzhou University of Chinese Medicine, Guangzhou, China; 6 Bendheim Integrative Medicine Center, Memorial Sloan Kettering Cancer Center, New York, NY USA; 7 State Key Laboratory of Dampness Syndrome of Chinese Medicine, the Second Affiliated Hospital of Guangzhou University of Chinese Medicine, Guangzhou, China

**Keywords:** Acupuncture, Gastric cancer, Adjuvant chemotherapy, Health-related quality of life, Symptoms

## Abstract

**Background:**

Patients with local gastric cancer experience a decline of Health-related quality of life (HRQOL) during adjuvant chemotherapy following gastrectomy. Our previous pilot study has indicated the potential of acupuncture to improve HRQOL and control cancer-related symptoms burden. This full-scale trial will focus on confirming the effect of acupuncture for patients with gastric cancer.

**Methods:**

A multicenter, open-label, three-arm randomized controlled trial with 249 patients will be conducted in China. Patients will be randomly assigned, in a ratio of 1:1:1, to receive high-dose acupuncture (HA, 7 times each chemo-cycle for 3 cycles), low-dose acupuncture (LA, 3 times each chemo-cycle for 3 cycles), or no acupuncture. The acupoints prescription consisted of bilateral ST36, PC6, SP4, DU20, EX-HN3, and selected Back-*shu* points. Patients-reported Functional Assessment of Cancer Therapy-Gastric (FACT-Ga) and modified Edmonton Symptom Assessment Scale (mESAS) during the therapy will be recorded. Area under curve (AUC, 21 days/cycle × 3 cycles) and average trajectory of FACT-Ga and mESAS will be calculated. The primary outcome will be the differences in AUC of the FACT-Ga Trial Outcome Index (TOI) between HA and LA versus control groups. Secondary outcomes include AUCs and average trajectory of other FACT-Ga subscales and mESAS scores.

**Discussion:**

This study aims to assess the effect of acupuncture and to compare the difference between LA and HA groups on HRQOL and symptom burden controlling in gastric cancer patients by an adequately powered trial.

**Trial registration:**

This study was approved by the Ethics Committee of the Guangdong Provincial Hospital of Traditional Chinese Medicine (approval number: BF2018-118) with registration at ClinicalTrials.gov (identifier: NCT04360577).

**Supplementary Information:**

The online version contains supplementary material available at 10.1186/s12955-023-02135-9.

## Background

Gastric cancer, the fifth most frequently diagnosed cancer and the third leading cause of cancer-related deaths in the world, still constitute a major global health problem, especially in China [1, 2]. Radical resection and postoperative adjuvant chemotherapy are possible ways to cure gastric cancer [3]. However, various symptoms caused by surgery and adjuvant chemotherapy [4, 5] seriously affect the health-related quality of life (HRQOL) and even lead to the interruption of chemotherapy, which increases the risk of tumor recurrence. Although numerous studies showed the impact of surgical technique on the long-term HRQOL [6–8], few studies showed the effect for the short-term HRQOL, especially within the first 3 months after surgery. While the most serious decline of HRQOL often happens within the first 3 months, with nausea, early satiety, reflux, and pain as the most common symptoms [9–11]. Thus, improving HRQOL during the period of adjuvant chemotherapy with an effective intervention has important clinical significance.

Acupuncture is the most widely used traditional and complementary medicine [12] and has been proven to be effective in reducing gastrectomy and chemotherapy-related symptoms by plenty of clinical practice and trials [13–18]. Some clinical practice guidelines and studies recommended acupuncture for cancer-related fatigue [19], cancer pain [20–22], nausea, vomiting [23], chemotherapy-induced peripheral neuropathy [24], hot flashes, and insomnia [25].

However, on the one hand, there are few large-sample randomized control trials or meta-studies on acupuncture improving the overall HRQOL of patients during chemotherapy in gastric cancer. On the other hand, the dosage of acupuncture, such as the number of treatment session, varies in different studies and impact the therapeutic effect [26].In the investigator’s pilot study (NCT 03753399), we presented preliminary evidence that acupuncture was associated with promising effects in improving HRQOL during adjuvant chemotherapy in gastric cancer patients, and showed a difference in the improvement of some symptoms, such as anxiety and depression, between the low-dose acupuncture group and the high-dose acupuncture group [27]. Therefore, studies with a larger sample size are needed to clarify the effect and optimal dose of acupuncture intervention to improve HRQOL of patients with adjuvant chemotherapy after radical gastrectomy.

In this study, we aim to design a multicenter, open-label, three-arm randomized controlled trial to fully assess the effect of acupuncture for HRQOL and symptoms with gastric cancer undergoing adjuvant chemotherapy (Aim 1) and compare the difference between low dose (LA) and high dose acupuncture (HA) groups (Aim 2).

## Methods

The EAGER study, acupuncture for improving the quality of life and symptoms in patients with gastric cancer undergoing adjuvant chemotherapy, follows the Helsinki Declaration and has been approved by the Ethics Committee of the Guangdong Provincial Hospital of Traditional Chinese Medicine (approval number: BF2018-118) with registration at ClinicalTrials.gov (identifier: NCT04360577) before the first participant enrollment. We followed the Standard Protocol Items: Recommendations for Interventional Trials [28] guideline for the reporting of the study protocol.

### Study design

**Fig. 1:**
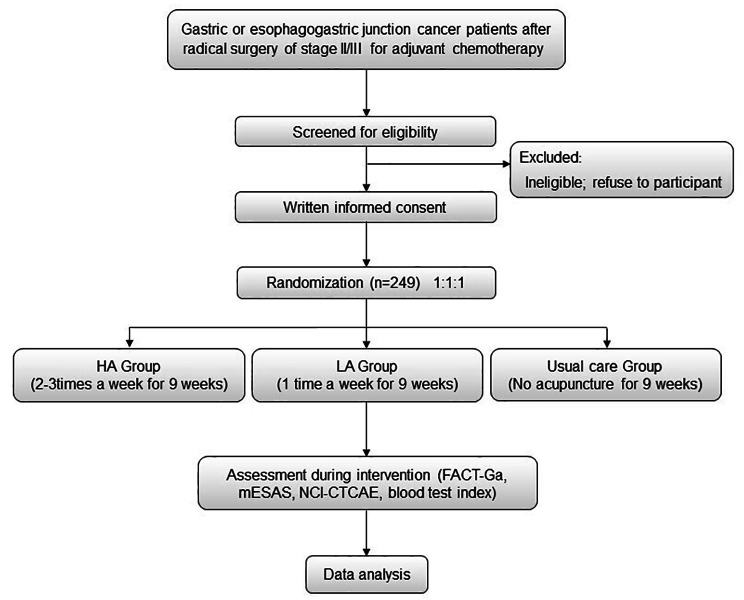
Trial procedure flowchart

A multi-center, three-arm, open-label randomized controlled trial will be conducted at 11 hospitals in China, including Guangdong Provincial Hospital of Traditional Chinese Medicine, the First Affiliated hospital of Sun Yat-sen University, Affiliated Hospital of Nanjing University of Traditional Chinese Medicine, the Sixth Affiliated Hospital of Sun Yat-sen University, Zhujiang Hospital of Southern Medical University, Guangdong Provincial People’s Hospital, Affiliated Cancer Hospital & Institute of Guangzhou Medical University, the First Affiliated Hospital of Guangzhou Medical University, the Fifth Affiliated Hospital of Sun Yat-Sen University, Foshan First People’s Hospital and Yuebei People’s Hospital. Participants will be recruited from the inpatient and outpatient departments of oncology and surgery. Eligible and consented patients will be randomly assigned, to receive high-dose acupuncture, low-dose acupuncture, or usual cancer with no acupuncture during the first 3 cycles of adjuvant chemotherapy after resection. Participants will be assessed at baseline and during the 3 cycles of chemotherapy (21 days for one cycle) post interventions. The design of the trial is outlined in Fig. 1.

### Participants

Patients meeting the following criteria are eligible: (1) pathologically diagnosed with gastric cancer or esophagogastric junction cancer after R0 resection and D2 lymph node dissection; (2) pathological stage II or stage III; (3) without tumor recurrence confirmed by image examination; (4) no chemotherapy after surgery, planning to accept at least 3 cycles of adjuvant chemotherapy; (5) age:18 ~ 75 years old; (6) ECOG score ≤ 2; (7) normal organ function, including (7.1) bone marrow function: absolute neutrophil count (ANC) ≥ 1.5 × 10e9/L, platelet (PLT) ≥ 100 × 10e9/ L, hemoglobin (Hb) ≥ 90 g/L; (7.2) kidney function: Serum creatinine (Scr) ≤ 1.5 mg/dl(133µmol/L), or creatinine clearance rate (Ccr) ≥ 60ml/min; (7.3) liver function: total bilirubin (TB) ≤ 1.5×upper limit of normal value (ULN), alanine transaminase (ALT) ≤ 2.5×ULN, aspartate transaminase (AST) ≤ 2.5×ULN; (8) can understand the study well and finish the questionnaires in this study;(9) with the written informed consent. Participants will be excluded if they meet any of the following criteria: (1) cannot finish the baseline assessment; (2) needle phobia; (3) currently diagnosed with a psychiatric disorder (e.g., severe depression, obsessive-compulsive disorder, or schizophrenia); (4) history of autoimmune diseases, hematological diseases or organ transplantation, or long term use of hormones or immunosuppressors; (5) implanted with heart pacemaker; (6) has accepted neoadjuvant radiotherapy before surgery; (7) planning to accept adjuvant radiotherapy after surgery; (8) with active infection; (9) acupuncture treatment within the previous 6 weeks; (10) pregnant or lactating women.

Research staff will screen eligible patients through physical examination and clinical tests. Eligible participants will be required to sign a consent form before enrolment and their information will remain confidential.

### Randomization

Patients will be randomly assigned to the HA group, LA group, and control group (1:1:1), using the central randomization system, allowing for full allocation concealment, provided by the Clinical Trial Management System of Xiyuan Hospital, China Academy of Chinese Medical Sciences (CACS). Random assignment will be stratified by resection extent (total/ proximal or distal gastrectomy) and neoadjuvant chemotherapy status (yes or no). An Electronic Data Capture (EDC) system provided by Xiyuan Hospital CACS will be used for research data management. The study coordinator at each site will ensure that the informed consent form has been obtained from each participant before randomization.

### Interventions

#### Chemotherapy

All participants will be treated with adjuvant chemotherapy with the CapeOx or SOX regimen. The CapeOx regimen consisted of 130 mg/m^2^ of oxaliplatin intravenously on the first day and 1000 mg/m^2^ of capecitabine twice daily for 14 consecutive days, every 21 days. The SOX regimen consisted of 130 mg/m^2^ of oxaliplatin intravenously on the first day and S-1 twice daily for 14 consecutive days, every 21 days. The dose of S-1 will also be calculated according to body surface area (BSA): BSA < 1.25m^2^, 80 mg/day; 1.25m^2^ ≤ BSA < 1.5m^2^, 100 mg/day; BSA > 1.5m^2^, 120 mg/day.

#### Selection of acupuncture points

The acupoints prescription has been developed based on the principles of Traditional Chinese Medicine (TCM) theory [29], literature evidence [30–33] and our clinical experience. It takes into account the gastrointestinal symptoms that are commonly experienced by gastric cancer patients undergoing chemotherapy after gastrectomy [9]. The acupuncture protocol including standard points and individualized points were proven feasible and acceptable to patients in the pilot trial. Standard points consist of bilateral Zusanli (ST 36), bilateral Neiguan (PC 6), bilateral Gongsun (SP 4), Baihui (DU 20), and Yintang (EX-HN3), The individualized points consist of several Back-*shu* points in the bladder meridian according to the meridian diagnosis of traditional Chinese medicine [27, 34]. Full information about the selection of Back-*shu* points is presented in the online supplementary appendix1. The total number of acupoints included is generally about 12 [27].

#### Methods of acupuncture in HA group and LA group

Patients will be in sitting, prone, or in a lateral position to accept acupuncture treatment. Sterile, disposable, stainless steel acupuncture needles (25 or 40 mm and 0.25 mm gauge; Hanyi Medical Instrument Co., Ltd, Beijing) will be gently inserted into acupuncture points. For ST36, PC6, and SP4, needles will be inserted perpendicular to a depth of approximately 10-30 mm from the skin surface. For DU20, EX-HN3, and Back-*shu* points, needles will be inserted obliquely 10-15 mm deep at an angle of 30 degrees to skin. Lifting and thrusting the needles to obtain the sensation known as “*De qi*”, a sense of soreness, warmth, tingling, or heaviness reported by patients suggested effective needling. An electric stimulator (G6805-1 EA apparatus; Xinsheng Industrial Co., Ltd, Qingdao, China) will be connected to the needle handles at bilateral ST36 and PC6 with a 2-HZ continuous wave, and the intensity of electric current will be increased up to a level that patient can perceive and tolerate. All needles will be retained for 20 min. All acupuncturists will be licensed with more than 3 years of clinical experience and receive training on the specific protocol.

Patients in the HA group will accept acupuncture treatment three times in the first week, and then twice a week in the next two weeks during each cycle of chemotherapy (21 days for 1 cycle) for three cycles, namely a total of 21 times acupuncture sessions; those in the LA group will take once a week for three cycles, namely a total of 9 times acupuncture sessions.

#### Methods of usual care in control group

Patients in the control group will only receive usual care according to NCCN clinical practice guidelines in oncology–Antiemesis [23] without acupuncture treatment. After the trial, they will receive the low-dose acupuncture treatment mentioned above for 3 cycles of chemotherapy for the sake of compensation according to their will.

#### Relevant concomitant care and interventions

In principle, all participants will not be given other traditional Chinese medicine therapies, such as Chinese herbs, moxibustion, and acupuncture apart from the study protocol. Receipt of any intervention outside the study protocol should be documented on case record form in detail.

### Outcomes

#### Primary outcome

The primary outcome will be the area under curve (AUC) of Functional Assessment of Cancer Therapy – Gastric(FACT-Ga) scale Trial Outcome Index (TOI) during the 3 cycles of chemotherapy.

#### Secondary outcomes

The secondary outcomes consist mainly of 7 indicators. **(1)** Average trajectory of FACT-Ga TOI over time; **(2)** Total AUC of FACT-Ga Scoring; **(3)** Average trajectory of FACT-Ga Scoring over time; **(4)** Total AUC of Gastric cancer subscale (GaCS) of FACT-Ga; **(5)** Average trajectory of GaCS of FACT-Ga; **(6)** Total AUC of Modified Edmonton Symptom Assessment Scale (mESAS); **(7)** Average trajectory of mESAS.

FACT-Ga has been widely used for the assessment of gastric cancer patients as a well-validated HRQOL instrument (https://www.facit.org/measures/FACT-Ga*)* [35, 36]. It contains four domains, physical well being (PWB) subscale, social/family well being (SWB) subscale, emotional well being (EWB) subscale, and functional well being (FWB) subscale that consists of 27 general HRQOL questions, plus a 19-item gastric cancer subscale, Gastric cancer subscale (GaCS). This is a symbol digital modality test in which the patient reports the intensity of related symptoms on a scale of 0 to 4. The higher scores are considered to be related to a better quality of life. The total score ranges between 0 and 184. FACT-Ga TOI is composed of PWB subscale, FWB subscale, and GaCS subscale of the FACT-Ga Scoring. The range of FACT-Ga TOI is 0-132. The higher score, the better quality of life. FACT-GA for each patient will be recorded at baseline and once a week during the 3 cycles of chemotherapy (21 days for one cycle).

ESAS is a questionnaire widespread used for symptom assessment in cancer patients [37–39]. It was composed of 10 items with a score range of 0–10 for each item, including pain, tiredness, nausea, depression, anxiety, drowsiness, appetite, wellbeing, shortness of breath, and others. ESAS was then modified in 2015 [40], with additional symptoms of constipation and sleep. The higher the score, the worse the symptom is. According to the data of our pilot study, a significant number of patients reported constipation and difficult sleeping, so we used a modified version of ESAS. mESAS scoring for each patient will be recorded every day in the first week, and then once a week in the next 2 weeks during the 3 cycles of chemotherapy (21 days for 1 cycle).

According to our pilot trial, the effect of EA should persist throughout the whole phase of the intervention, rather than at any specific time point [27]. Therefore, the standardized area under curve (AUC, 21days/cycle×3 cycles), calculated by linear interpolation, will be used to reflect the repeated measures of HRQOL (FACT-Ga) and symptom burden (mESAS) during the intervention period [41, 42]. Furthermore, we hypothesized that the effect of EA will be mainly reflected on the TOI scores, which regards more with gastric cancer.

The other pre-specified outcomes include blood test indexes (concentration of circulating myeloid-derived suppressor cells, concentration of circulating Treg cells, and the number of circulating tumor cells), the incidence of treatment-emergent adverse events defined as any adverse events happened after randomization. The severity is validated using national cancer institute common terminology criteria for adverse events (NCI-CTCAE) V4.

The outcome assessments will be performed by outcome assessors at the inpatient department of each participating site. All the outcome assessors will be trained in conducting interviews and performing measurements before the study begins and will follow a standard protocol. The schedule of measurements is presented in Table 1.

**Table 1:** Measurements to be taken at each point in the trial

Period		Screening	1st cycle	2nd cycle	3rd cycle	4-6th cycle
Timeline		D-21-0	D1-21	D1-21	D1-21	
**Enrolment**		**×**				
**Activity**						
MPHTJP test^a^		**×**				
conventional treatment			**×**	**×**	**×**	**×**
Acupuncture(LA/HA group^b^)			**×**	**×**	**×**	
Compensatory acupuncture (Control group)						**×**
**Assessment**						
HRQOL^c^	FACT-Ga^d^		**×**	**×**	**×**	
Symptoms	mESAS^e^		**×**	**×**	**×**	
AEs/SAEs^f^	NCI-CTCAE^g^ V4.0		**×**	**×**	**×**	
Blood test indexes	MDSCs^h^	**×**			**×**	
	Tregs^i^					
	CTC^j^					

^a^MPHTJP test: Measuring pain heat threshold of Jing point and presented in the online supplementary appendix

^b^LA/HA group: Low dose acupuncture/High dose acupuncture group

^c^HRQOL: Health-related quality of life

^d^FACT-Ga: Functional Assessment of Cancer Therapy – Gastric scale

^e^mESAS: Modified Edmonton Symptom Assessment Scale

^f^AEs/SAEs: Adverse events/Severe adverse events

^g^NCI-CTCAE: National cancer institute common terminology criteria for adverse events

^h^MDSCs: Myeloid-derived suppressor cells

^i^Tregs: Treg cells

^j^CTC: Circulating tumor cells

### Safety assessment

Although acupuncture is a relatively safe treatment with a low risk of adverse events [43], any adverse events (AEs) during the study period will be assessed, treated, and documented in the case report form (CRF). AEs, such as broken needle, localized hematoma, bleeding, or localized severe sharp pain will be categorized as acupuncture treatment-related; AEs such as chemotherapy-related or surgical treatment-related will be categorized as non-acupuncture treatment-related. Severe adverse events (SAEs) occurred in any of the three groups would be reported to the independent ethics committee. The ethics committee will conduct an annual follow-up review of this study.

### Quality control

To ensure consistency of the study, research staff in recruitment sites will receive extensive training from the principal investigator (HBZ) concerning the protocol, manipulating methods of acupuncture and follow-up of participants. Additionally, the same participants should be taken charge of by the same research assistant/outcome assessor throughout the trial. They will instruct participants to complete HRQOL and symptoms questionnaires, explain the contents of questionnaires if necessary and remind the participants of their schedule by phone or WeChat. On each outcome assessment visit, the forms will be collected and examined by outcome assessors and recorded in CRF in time.

### Data management

All the data collected will be documented in CRF first by clinical research coordinators and checked by 2 trained clinical research associates before double data entry into EDC system by trained research assistant. This rule is set to ensure the data is accurate, authentic, and traceable. The database will be protected by a password, and only the principal investigator will have access to the final dataset. A data monitoring committee that is independent of researcher and sponsor and has no conflict of interest with this study will be estimated. It will be responsible for the management of data quality.

### Sample size

In our pilot trial, the total AUCs of FACT-Ga TOI during the 3 cycles of chemotherapy were 5402 and 4780 in the acupuncture and control groups, respectively, with a standardized derivation of 1109 [27]. Thus, a total of 62 participants will be needed in each arm to detect a significant difference between the control group and either LA or HA group, with an α of 0.025 and a power of 80%. Considering the total dropout rate of 22.41% in our pilot study and eliminating the impact of COVID-19 epidemics, we plan to enroll a total of 249 patients to allow a dropout rate of 25%.

### Statistical analysis

Full Analysis Set (FAS): FAS will be composed of all randomized subjects according to the intention-to-treat analysis (ITT) principle, excluding those with the following criteria: (1) patients who request to withdraw from the study and provide an informed statement that he/she does not want to be analyzed, (2) patients who do not meet the inclusion/exclusion criteria incorrectly included when random, and (3) patients who are only with baseline data, without any data after chemotherapy in this trial.

Per-protocol Set (PPS): PPS will be composed of patients who complete at least 50% treatment according to the protocol which is defined as: (1) no less than 3 cycles of chemotherapy; (2) no less than 2 cycles of acupuncture treatment with no less than 50% acupuncture treatment for per cycle (i.e., no less than 4 times acupuncture in HA group and 2 times acupuncture in LA group);

FACT-Ga and mESAS scales score, demographics data, baseline clinical characteristics, the other efficacy indicators and safety indicators will be analyzed and compared in FAS. Sensitivity analysis of FACT-Ga and mESAS scales score will be used in PPS.

Numerical data are expressed as proportions, rates and frequencies. Continuous data are presented as the mean standard deviation for normal distribution, or the median and 25 ~ 75% percentile for other distributions.

We will modify our analyses to compare the outcomes in the FAS population; we will also compare the differences in the PPS population as sensitivity analysis. Missing data will be imputed using the worst observation carried forward (WOCF) method to calculate the AUC. We will use student’s t-test, analysis of variance (ANOVA) with least significant difference (LSD) for post hoc test and baseline score as covariate, χ2 test, or Fisher’s exact test to test the differences among groups. In addition, the average and the worst score of the FACT-Ga and mESAS scale during the chemotherapy will use the same statistical method. Demographics data, baseline clinical characteristics, and the other outcomes will be reported and compared using χ2 test or Fisher’s exact test. All hypothesis tests in this study are 2-sided tests, with an alpha < 0.05. Data will be analyzed using SPSS 17.0 and Stata 11.0 software.

## Discussion

Patients with postoperative adjuvant chemotherapy for gastric cancer have poor quality of life and heavy symptom burden. In clinical practice, there is no effective treatment to solve this problem. Acupuncture has been proven to be effective in improving side effects associated with various tumor treatments, and it is worth conducting large-scale clinical studies to clarify the efficacy of acupuncture in improving overall quality of life in these patients.

A sufficient acupuncture dose, especially the number of treatments, is important for the clinical effectiveness of acupuncture treatment for common conditions. Some studies reported that more acupuncture sessions appeared to be associated with better outcomes in patients with knee osteoarthritis and chronic pain [44, 45]. However, excessive acupuncture treatment may lead to increased medical resources, heavier traveling, and financial burden. The frequency of acupuncture in oncology settings varies from daily [46] to weekly [18] with huge heterogeneity in different countries and regions. In many Chinese studies, 2–3 times a week was the common choice for the frequency of acupuncture interventions [15, 47], whereas weekly acupuncture was more common in the US [18, 48]. The results of the pilot trial [27] showed a similar trend in quality of life scores between the high-dose and low-dose groups of acupuncture. In this full-scale trial, if the similarity between LA and HA groups is still observed, an equivalence study between high-dose and low-dose acupuncture will be worthy, considering the transportation, economic, and healthcare cost of high-dose acupuncture. Alternatively, if an obvious trend of differentiation is observed, then a differential study may be worth conducting to determine the appropriate dose of acupuncture.

This study will use AUCs as the primary endpoint instead of the scores at different time points during the postoperative adjuvant chemotherapy period to decrease heterogeneity. The AUC, as a standard metric in pharmacokinetic analyses, is often used for describing drug concentration. It also has been suggested as a preferred statistic in a variety of clinical settings [41, 42, 49, 50] for embracing all available information to interpret how much improvement after treatment, including evaluating pain relief and enhancement of sleep quality over time [51, 52]. The AUC provides relevant information on how much more improvement in HRQOL, on average, a patient may achieve with acupuncture therapy compared with usual care, rather than the scores at any specific time point.

This study continued to use an open-label design approach, considering the controversy about sham acupuncture control and the clinical meaning of acupuncture. Although sham-controlled trials with blinding are preferred from the perspective of methodology, trials without sham acupuncture have also been recommended recently, because of their emphasis on the practical applicability and increased external validity over treatment efficacy [53]. Sham acupuncture also has some limitations including latent ethics issues brought by unnecessary transportation costs for the sham acupuncture group, uncertain effect of inert control [54, 55], and the lack of consensus on approaches. For these reasons, more and more trials did not include a sham-acupuncture control group [48, 56]. We agree with the opinion of Molassiotis A. and colleagues that shifting the focus from placebo-related issues to those with clinical meanings is appropriate [57]. Although we did not use sham acupuncture in the pilot trial, the differences in quality of life between acupuncture and control groups are over the minimally important difference (MID) [35], indicating that this benefit is clinically meaningful. In addition, in order to control bias as much as possible, the statistician will be blinded to the treatment in this trial.

This study will be the first large-sample, multi-center randomized controlled trial on the efficacy of acupuncture on HRQOL and symptoms during postoperative adjuvant chemotherapy for gastric cancer. We are going to assess the effect of acupuncture, and to compare the difference between LA and HA groups on HRQOL in gastric cancer patients in a rigorous way by an adequately powered trial.

## Electronic supplementary material

Below is the link to the electronic supplementary material.

Supplementary Material 1

## Data Availability

Not applicable.

## Declarations

### Trial situation

The trial is currently in the recruitment phase. This protocol version is V2.

## Abbreviations

HRQOL: Health-related quality of life
HA: High-dose acupuncture
LA: Low-dose acupuncture
FACT-Ga: Functional Assessment of Cancer Therapy-Gastric scale
PWB: Physical well being subscale of FACT-Ga
SWB: Social/family well being subscale of FACT-Ga
EWB: Emotional well being subscale of FACT-Ga
FWB: Functional well being subscale of FACT-Ga
GaCS: Gastric cancer subscale of FACT-Ga
TOI: Trial Outcome Index, composed of PWB subscale, FWB subscale, and GaCS subscale of the FACT-Ga Scoring
mESAS: Modified Edmonton Symptom Assessment Scale
AUC: Area under curve
AEs: Adverse events
SAEs: Severe adverse events
EDC: Electronic Data Capture
NCI-CTCAE: National cancer institute common terminology criteria for adverse events
