# Supplementary material for: Electro-acupuncture for health-related quality of life and symptoms in patients with gastric cancer undergoing adjuvant chemotherapy (EAGER): a protocol for a multicenter randomized controlled trial
Source: Health Qual Life Outcomes. 2023 Jul 11;21:70. doi: 10.1186/s12955-023-02135-9 (PMC10334569; doi:10.1186/s12955-023-02135-9)
Supplement: Supplementary file 1 — Supplementary Material 1 [file 12955_2023_2135_MOESM1_ESM.docx]

**Methods of** **measuring pain heat threshold of Jing point (MPHTJP) and selection of back-*shu* points**

A. Method of measuring pain heat threshold of *Jing* point: the specific operation process should be informed to the patient before measuring. After igniting the joss-stick(Xiang Tibaten joss-stick, Gucheng, Baoding, China), then fix the joss-stick on a stand first, keep the joss-stick in a horizontal direction, 2mm away from the *Jing* point until the patient senses a burning pain. The time for this heat threshold (beat, timed by Soundcorset APP,120 beats/ min) will be recorded. The measuring *Jing* points include *ShaoShang* (LU11), *ShangYang* (LI1), *ZhongChong* (PC9), *GuanChong* (TE1), *ShaoChong* (HT9), *ShaoZe* (SI1), *YinBai* (SP1), *Dadun* (LR1), *LiDui* (ST45), *ZuQiaoYin* (GB44), *NeiZhiYin* (KI-1’), *ZhiYin (*BL67). All *Jing* points are bilateral. The order of testing is from hand to foot, from left to right.

B. Location of *Jing* points: (Figure S1)


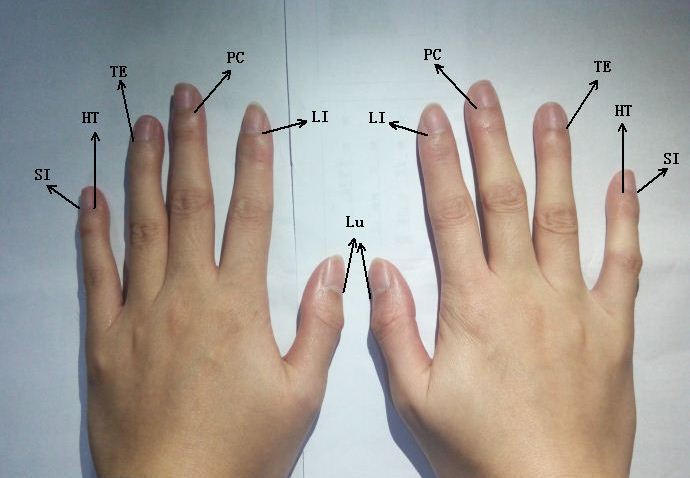

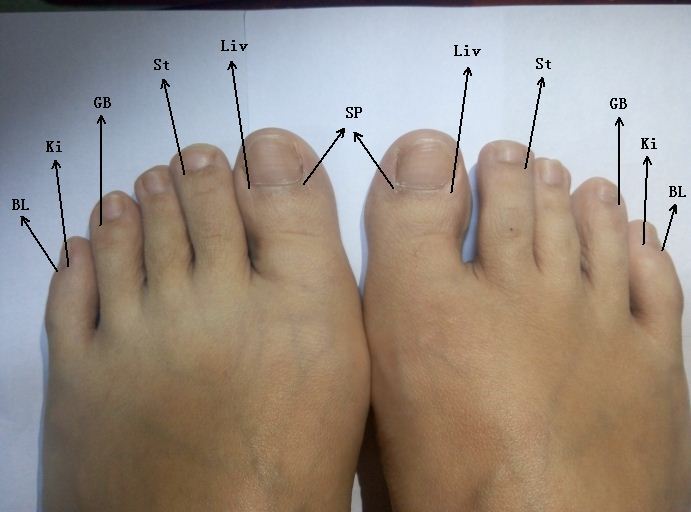


Figure S1-1 *Jing* point’ location on hands Figure S1-2 *Jing* point’ location on foots

*The human foot and hands in Figure S1 belong to the first author Dr. Xue-song Chang.

C. Selection of Back-*Shu* points: two research staff are needed to complete the operation. One does the point heating, the other one records measure time of *Jing* point’s painful heat threshold in table1 and calculates the mean and standard deviation. If the value of one *Jing* point test time is more than the mean plus standard deviation, mark *Xu* (deficiency); conversely, if less than the mean minus standard deviation, mark *Shi* (excess). Then, the corresponding to those Xu (deficiency) Jing point meridian Back-*Shu* points will be selected as the points for acupuncture group treatment. (Table S1)

Table S1 Measure time of pain heat threshold of *Jing* point (beat)

| No. of  centrum | *Jing* point  (Back-*Shu* point) | Left hand | Right hand | No. of  centrum | *Jing* point  (Back-*Shu* point) | Left foot | Right foot |
| --- | --- | --- | --- | --- | --- | --- | --- |
| 3 | LU11 (BL13) |  |  | 11 | SP1 (BL20) |  |  |
| 16 | LI1 (BL25) |  |  | 9 | LR1 (BL18) |  |  |
| 4 | PC9 (BL14) |  |  | 12 | ST45 (BL21) |  |  |
| 13 | TE1 (BL22) |  |  | 10 | GB44 (BL19) |  |  |
| 5 | HT9 (BL15) |  |  | 14 | KI-1’ (BL23) |  |  |
| 18 | SI1 (BL27) |  |  | 19 | BL67 (BL28) |  |  |
| mean/standard deviation value of hand: ____/____ | | | | mean/standard deviation value of foot: ___/____ | | | |
| Selection of Back-*Shu points* | | |  | | | | |
